# Relationship between awareness of cervical cancer and HPV infection and attitudes towards HPV vaccine among women aged 15-49 years: a cross-sectional study

**DOI:** 10.1590/1516-3180.2021.0145.27072021

**Published:** 2022-04-02

**Authors:** Engin Yurtçu, Reyhan Aydın Doğan, Büşra Karaaslan, Sibel Mutlu

**Affiliations:** I PhD. Assistant Professor, Department of Gynecology and Obstetrics, Faculty of Medicine, Karabük University, Karabük, Turkey.; II PhD. Assistant Professor, Department of Midwifery, Faculty of Health Sciences, Karabük University, Karabük, Turkey.; III BSc. Midwife, Department of Gynecology and Obstetrics, Kocaeli Derince Training and Research Hospital, Izmir, Turkey.; IV PhD. Gynecology and Obstetrics Specialist, Department of Gynecology and Obstetrics, Private Yüzyıl Hospitals, Pendik, Istanbul.

**Keywords:** Papillomavirus vaccines, Uterine cervical neoplasms, Papillomavirus infections, HPV vaccine, Cervical cancer, Uterine cervical cancer, HPV infection, Human papillomavirus

## Abstract

**BACKGROUND:**

Cervical cancer is a type of cancer caused by human papillomavirus (HPV).

**OBJECTIVE:**

To determine the relationship between awareness of cervical cancer and HPV infection and attitudes towards HPV vaccine among women aged 15-49 years.

**DESIGN AND SETTING:**

Cross-sectional study conducted at Karabük Training and Research Hospital, Turkey.

**METHODS:**

500 women who visited the gynecology outpatient clinic of a public hospital between July 15 and December 31, 2019, were selected through random sampling. Data were collected using a sociodemographic questionnaire comprising nine questions (created by the researchers), the HPV and Cervical Cancer Awareness Questionnaire and the Carolina HPV Immunization Attitudes and Beliefs Scale.

**RESULTS:**

The relationship between the awareness questionnaire and the beliefs scale was explained through simple effect modeling of a structural equation. The women’s knowledge score regarding cervical cancer and HPV infection was 4.69 ± 4.02 out of 15. Women were afraid of being diagnosed with cervical cancer and HPV infection, but they did not have sufficient information. They had poor information about the HPV vaccine, did not know how to obtain the vaccine and did not have enough information about its benefits and harmful effects. Women who were afraid of getting cervical cancer, and who thought that they were at risk, had more information about the HPV vaccine.

**CONCLUSION:**

Women need information about cervical cancer, HPV infection and the HPV vaccine. Midwives, nurses and physicians who provide healthcare services in gynecological follow-ups should provide information to women about the HPV vaccine and cervical cancer.

## INTRODUCTION

Although the incidence of cervical cancer has decreased in developed countries over recent decades through effective screening programs, it continues to be an important health problem, especially in developing countries. With 569,847 new cases and 311,365 deaths worldwide in 2018, cervical cancer is expected to be the fourth most common type of cancer and the fourth leading cause of cancer death among women. Out of these deaths, 90% occur in underdeveloped or developing countries. In 2018, 2,356 new cases and 1,280 deaths related to cervical cancer were seen in Turkey.^1^

Human papillomavirus (HPV) is the most common sexually transmitted infection.^2^ It has been estimated that the lifetime probability of acquiring HPV exceeds 80% among women and 90% among men.^3^ Different groups of HPVs exist, with different epithelial tropisms (cutaneous and mucosal) and life-cycle strategies. Many HPVs are classified as low risk (lrHPV) because they are very rarely associated with neoplasia or cancer in the general population. These lrHPVs typically cause indeterminate/undetectable infections or benign papillomas that can last for months or years but are eventually cleared by the host’s immune system. High-risk HPV (hrHPV) types are the cause of many major human cancers, including almost all cases of cervical cancer, a large proportion of other anogenital cancers and an increasing number of head and neck tumors.^4^ HPV infections can be temporary or permanent. Most cervical HPV infections (around 90%) are cleared by cell-mediated immunity within one to two years of exposure. In lrHPV infections, clearance occurs within a shorter period than in hrHPV infections. Among all HPV infections, 5%-10% cause persistent disease.^5^

Cervical cancer has a long preinvasive period due to lesions associated with persistent hrHPV infection. Early diagnosis of these preinvasive lesions using screening methods (HPV DNA tests, cervical cytological tests, etc.), effective treatment of these lesions and administration of HPV vaccines can prevent this disease. Most cases of cervical cancer occur in women who have never been screened or were screened poorly.^4,5^

HPV vaccination has the potential to greatly reduce the morbidity and mortality associated with genital HPV infections and is recommended by the American Society of Obstetrics and Gynecology (ACOG) for all women and men aged 9-26 years.^6^ As cervical cancer has a long preinvasive period, it can be diagnosed and treated early by means of the screening programs that have been developed. For this reason, evaluating society’s attitudes and beliefs about cervical cancer and HPV vaccine and increasing the level of knowledge are important in terms of preventive medical practices.

Greater awareness among sexually active women aged 15-49 years regarding cervical cancer will decrease the rate of occurrence of this disease and increase the levels of knowledge about HPV vaccines and the vaccination rate.^6,7,8,9,10^

## OBJECTIVE

The aim of this study was to determine the relationships between knowledge of cervical cancer, awareness of HPV infection and attitudes towards HPV vaccines among women aged 15-49 years.

## METHODS

### Research type and sampling

This cross-sectional study was planned with the aim of determining the levels of knowledge about HPV and cervical cancer, levels of knowledge regarding preventive measures, health-related beliefs and awareness about HPV vaccines among women aged 15-49 years. The study population comprised women (28,356 women) within this age group who were living in the province of Karabük, Turkey.^11^ However, the sample used in this study comprised 500 women, as calculated through G-power analysis with a 95% confidence interval and 5% margin of error, assuming 75.2% prevalence. Data were collected at the gynecology and diseases outpatient clinic of a public hospital between July 15 and December 31, 2019.

### Data collection tools

To collect data, a questionnaire on sociodemographic characteristics comprising nine questions was prepared in line with the literature by the researchers. In addition, the HPV and Cervical Cancer Awareness Questionnaire and the Carolina HPV Immunization Attitudes and Beliefs Scale (CHIAS) were used.(12,13) Permission for this study was obtained from the Bülent Ecevit University Human Research Ethics Committee (dated June 27, 2019; approval no. 600) and from the institute at which this research was conducted. After obtaining permission from the women who agreed to participate in the study, data were collected through face-to-face interviews. The study was conducted in accordance with the Declaration of Helsinki.

### Evaluation of data

The statistical analysis for this study was done with the aid of the SPSS 20 computer software (SPSS, Chicago, United States). Given that the skewness and kurtosis values of the data remained within the +2.0/-2.0 range limit, the data were considered to follow normal distribution.^14^ Computer-assisted data analysis was used for the basic evaluation (correlations and frequencies) on the study data. We found that relationships between pairs of scales were explained through first-order factor analysis. A computer-assisted analysis program was used for factor analysis. Pearson’s correlation analysis was used to determine the relationships between scales and subdimensions.^15^ The data obtained were evaluated with a 95% confidence interval and a significance level of P < 0.05.

## RESULTS

The Cronbach’s alpha coefficient of the awareness scale for cervical cancer and HPV infection was determined as 0.91 by Ingledue.^12^ In the validity-reliability study for use of this awareness scale in Turkish, conducted by Özdemir and Kısa, Cronbach’s alpha coefficient was 0.71.^16^ In our study, the Cronbach’s alpha coefficient of the awareness scale for cervical cancer and HPV infection was 0.81. The Cronbach’s alpha coefficient of the CHIAS was determined by McRee et al.^17^ Accordingly, the alpha values for “harm”, “obstacles”, “effects” and “uncertainty” were 0.69, 0.69, 0.61 and 0.66, respectively.^17^ Cronbach’s alpha was 0.62 in the validity-reliability study for use of the Carolina HPV Immunization Attitudes and Beliefs Scale in Turkish, conducted by Sunar and Süt.^18^ In our study, the Cronbach’s alpha of the CHIAS was 0.80; the alpha values for the subdimensions “harm”, “obstacles”, “effects” and “uncertainty” were 0.73, 0.75, 0.74 and 0.70, respectively.

The sociodemographic characteristics of the women participating in the study (n = 500) are shown in Table 1. The mean age of the participants was 23.52 ± 5.656 years, and the mean number of sexual partners was 0.22 ± 0.529. Among these women, 78.6% were high school graduates (n = 393), 79% were single (n = 395) and 50.6% (n = 253) had a monthly income of between 0 and 500 Turkish lira (approximately 0 to 70.32 United States dollars). In addition, 63.6% (n = 318) had health insurance from the Turkish Social Insurance Institution (SGK) and 95% (n = 485) had not reached the menopause (Table 1).

**Table 1.** Sociodemographic characteristics of the women participating in the study (n = 500)

	n (%)
**Age in years**	23.52 (5.656)*
**Education level**	
Illiterate	4 (0.8)
Literate	15 (3)
Primary school graduate	21 (4.2)
Secondary school graduate	18 (3.6)
High school graduate	49 (9.8)
University/college level	393 (78.6)
**Marital status**	
Single	395 (79)
Married	91 (18.2)
Widow	5 (1)
Significant partnerships	8 (1.6)
In-home or out-of-home partnerships	1 (0.2)
**Monthly income**	
0-500 TL	253 (50.6)
501-1000 TL	111 (22.2)
1001-1500 TL	22 (4.4)
1501-2000 TL	26 (5.2)
2001-2500 TL	30 (6)
2501-3000 TL	21 (4.2)
Over 3000 TL	37 (7.4)
**Health insurance**	
Social insurance institution	318 (63.6)
Pension fund	60 (12)
Pension fund for the self-employed (Bağ-kur)	65 (13)
Social security institution	43 (8.6)
Optional insurance	9 (1.8)
Unemployment insurance	5 (1)
**Menopausal status**	
Yes	6 (1.2)
No	485 (97)
Not sure	9 (1.8)

*Mean (standard deviation); TL = Turkish lira.

### Levels of knowledge regarding HPV and cervical cancer

The mean knowledge score from the first 15 questions on the awareness scale for cervical cancer and HPV infection was 4.69 ± 4.02. More than half of the women provided incorrect answers to the information questions about cervical cancer and HPV infection in the first section. At the same time, the mean knowledge scores were also low.

### The perceived threat due to cervical cancer

Regarding the perceived threat due to cervical cancer, perceived sensitivity and severity scores, which are subdimensions of health beliefs, were evaluated. The subdimension score for perceived sensitivity was determined to be a maximum of 45.^12,16^

### Perceived sensitivity

The mean perceived sensitivity score in our study was 25.32 ± 6.38. Among the participants, 50% were worried about getting cervical cancer and HPV. Conversely, 50% of the women stated that they did not have any information about prevention of cervical cancer and the measures to be taken against HPV infection.

### Perceived severity

Similarly, the mean perceived severity score was 17.74 ± 4.03. The perceived severity scores of the participants ranged from 6 to 30 points. Among the women, 41.2% saw HPV infection as a life-threatening disease, whereas 38.2% saw cervical cancer as a curable disease.

For each item derived from the previous versions of the CHIAS, the expressions were changed to reflect the perspective of a young adult rather than a parental perspective. In this process, the sentence format used by Dempsey et al. was taken as an example.^19^ The subdimension scores of the CHIAS were as follows: harm = 13.8 ± 3.37; obstacles = 8.53 ± 1.97; effects = 4.61 ± 1.30; and uncertainty = 4.42 ± 21.22.

In our study, the relationships between the subdimensions of the HPV and cervical cancer awareness questionnaire and those of the CHIAS were explained through simple effect modeling from a structural equation model (SEM). The adaptation values were as follows: minimum discrepancy (CMIN) = 34.911; degrees of freedom (df) = 13; minimum discrepancy/degrees of freedom ratio (CMIN/df) = 2.685; root mean square error of approximation (RMSEA) = 0.058; comparative fix index (CFI) = 0.938; and goodness-of-fit index (GFI) = 0.981. Because CMIN/df was not within the required limits, correction indices were examined. The “effects” subdimension of CHIAS provided a correction in accordance with the modification index, with the item of levels of knowledge of cervical cancer and HPV: the F1 and F2 dimensions.

The analysis was repeated by removing the “effects” subdimension item from the model. Then, the adaptation values were as follows: CMIN = 8.617; df = 8; CMIN/df = 1.077; RMSEA = 0.012; CFI = 0.998; and GFI = 0.994. All of the adaptation criteria were thus met within the desired limits.

The nonstandard path coefficient of F2 was 0.152, and this was statistically significant (P < 0.001). The standard path coefficient for this item was 0.249. The nonstandard path coefficient of the perceived severity subdimension was 1, and this was statistically significant (p < 0.001). The standard path coefficient for this item was 0.851. The nonstandard path coefficient of the perceived sensitivity subdimension was 1.366, and this was statistically significant (P < 0.001). The standard path coefficient for this item was 0.736.

**Figure 1.**
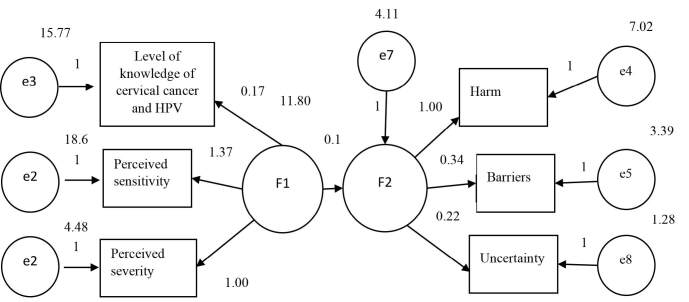
Nonstandard path coefficients between the HPV and cervical cancer awareness questionnaire and the Carolina HPV Immunization Attitudes and Beliefs Scale (CHIAS).

The nonstandard path coefficient of the “harm” subdimension was 1, and this was statistically significant (P < 0.001). The standard path coefficient for this item was 0.62. The nonstandard path coefficient of the “obstacles” subdimension was 0.336, and this was statistically significant (P < 0.001). The standard path coefficient for this item was 0.357. The nonstandard path coefficient of the “uncertainty” subdimension was 0.223, and this was statistically significant (P < 0.001). The standard path coefficient for this item was 0.382. The nonstandard path coefficient for the levels of knowledge of cervical cancer and HPV was 0.174, and this was statistically significant (P < 0.001). The standard path coefficient for this item was 0.149 (Figure 1; Table 2).

CMIN = minimum discrepancy; df = degrees of freedom; CMIN/df = minimum discrepancy/degrees of freedom ratio; RMSEA = root mean square error of approximation; GFI = goodness-of-fit index; AGFI= adjusted goodness-of-fit index; CFI = comparative fix index; TLI = Turker-Lewis index.

**Table 2.** Structural equation model (SEM) analysis between the HPV and cervical cancer awareness questionnaire and the Carolina HPV Immunization Attitudes and Beliefs Scale (CHIAS)

Scale subdimensions and factor two	Impact direction	Factors	β^0^	β^1^	Std error	Statistical test	P	R^2^
F2	<---	F1	0.249	0.152	0.051	2.981	0.003	0.062
Perceived severity	<---	F1	0.851	1				0.725
Perceived sensitivity	<---	F1	0.736	1.366	0.3	4.559	< 0.001	0.542
Harm	<---	F2	0.62	1				0.384
Barriers	<---	F2	0.357	0.336	0.099	3.401	< 0.001	0.128
Uncertainty	<---	F2	0.382	0.223	0.065	3.421	< 0.001	0.146
Level of knowledge about cervical cancer and HPV	<---	F1	0.149	0.174	0.064	2.729	0.006	0.022

β^0^ = standardized coefficient; β^1^ = non-standardized coefficient; Std error = standard error; R^2^ = coefficient of determination.

Pearson’s correlation was used because it showed parametric distribution between the awareness scale for cervical cancer and HPV infection and the subdimensions of the CHIAS (Table 3). There were no relationships between the subdimension of levels of knowledge of cervical cancer and HPV of the awareness scale for cervical cancer and HPV infection and the “harm”, “obstacles” and “uncertainty” subdimensions of the CHIAS. On the other hand, a positive and highly significant relationship was found with the “effects” subdimension. Very high positive correlations were found between the perceived sensitivity subdimension of the awareness scale for cervical cancer and HPV infection and the “harm”, “effects” and “uncertainty” subdimensions of the CHIAS. Moreover, very high significant positive correlations were found between the perceived severity subdimension of the awareness scale for cervical cancer and HPV infection and the “harm”, “effects” and “uncertainty” subdimensions (Table 3).

**Table 3.** Correlation between the awareness scale for cervical cancer and HPV infection and the subdimensions of the Carolina HPV Immunization Attitudes and Beliefs Scale (CHIAS)

n = 500	CHIAS subdimensions							
	Harm		Barriers		Effects		Uncertainty	
	r	P	r*	P	r*	P	r*	P
**Level of knowledge of cervical cancer and HPV**	-0.83	0.65	-0.06	0.88	0.20**	0.00	0.04	0.31
**Perceived sensitivity**	0.12**	0.00	0.06	0.12	0.15**	0.00	0.10*	0.02
**Perceived severity**	0.12**	0.00	0.06	0.12	0.12**	0.00	0.09*	0.04

*Correlation is significant at the 0.05 level; **Correlation is significant at the 0.01 level.

## DISCUSSION

Cervical cancer is a type of cancer that is monitored regularly through screening programs around the world, including in Turkey, and it can be treated quickly when detected.^20,21^ Through use of these screening programs for cervical cancer, mortality and morbidity due to this disease are gradually decreasing.^21^ The important factors in treating this disease are early diagnosis and women’s awareness. The degree of cancer or the size of the lesions that can be treated are decisive in the treatment process.^20,22^ Although cervical cancer is preventable, reports in the literature have demonstrated that, despite knowing about cervical cancer, women are not aware of the factors involved in its development.^20,23,24^

In our study, the women’s knowledge score about cervical cancer and HPV infection was low (4.69 ± 4.02), contrary to the findings in studies in the literature. Although 50% of the women were afraid of getting cervical cancer and HPV infection, they did not have any information about preventing this infection. In a study conducted by Montgomery et al. to determine the level of knowledge of cervical cancer and HPV, the knowledge score was 7.39 among 149 women.^23^ In a study by Ozan et al., 336 women who visited a gynecology outpatient clinic were assessed regarding their level of knowledge of cervical cancer and HPV, and it was observed that although 86.6% of them knew about cervical cancer, only 33.6% knew about HPV infection.^24^ Pehlivanoğlu et al. found that 26.8% out of 295 women who visited a family medicine outpatient clinic had never heard of the Pap smear test and 43.4% did not know about HPV infection.^20^

Although the women in our study had heard about HPV vaccines, their attitudes and knowledge regarding HPV vaccination was inadequate. This situation might have originated from the women’s low awareness of cervical cancer and inadequate knowledge of HPV infection. The level of knowledge of HPV vaccines that we found in our study was consistent with data in the literature.^9,13,25^

Pelullo et al. conducted a study among 556 nursing students, with the aim of examining their knowledge and attitudes regarding HPV vaccines. They found that although almost all the students had heard about the vaccine, only 36.5% were aware of its risk factors.^13^

In a study by Yılmaz et al., in which 624 nursing students were examined in terms of their knowledge, behavior and attitudes in relation to HPV vaccines, their levels of knowledge regarding the vaccine were lower than their levels of knowledge regarding HPV infection.^9^ Although 87.7% of those students knew that an HPV vaccine for women exists, only 52.4% were aware of the existence of an HPV vaccine for men.^9^

In our study, the subdimension scores for “obstacles” “harm” and “uncertainty” in relation to getting the HPV vaccine were low. This might have been due to lack of knowledge about the vaccine, lack of vaccine availability, the women’s lack of awareness about the vaccine effects and unavailability of the vaccinees. This result was consistent with data in the literature.^9,13,26^

In our study, significant relationships were found between the responses to the questionnaire regarding levels of knowledge of cervical cancer and HPV infection and the subdimensions of the CHIAS. Women who were worried about cervical cancer had higher levels of knowledge about HPV. Meanwhile, a significant relationship was found between women who thought they had a high probability of getting cervical cancer or HPV infection and the total knowledge score and the “harm” subdimension score. There were significant relationships between women’s fear of being infected with HPV virus and the “effects” and “obstacles” subdimension scores of the CHIAS. This might have been due to inability to cover the cost of the vaccine, lack of a vaccination program across the country and lack of information about where women can obtain the vaccine. This result was consistent with data in the literature.^9,13^

Knowledge about HPV vaccines increased with the perceived sensitivity, and there was a positive relationship between them. Conversely, a negative relationship was observed between the level of knowledge of cervical cancer and HPV infection and the “harm” subdimension. As the level of knowledge increased, the number of women thinking that the vaccine was harmful decreased. These results were consistent with data in the literature.^9,10,13,27^

Pelullo et al. reported that there was a positive relationship between the levels of knowledge of HPV vaccines among the students in their study and these students’ awareness of the risk factors.^13^

Giuseppe et al. explored HPV awareness among 1,348 adolescent girls and young women and reported that those who saw themselves at risk of cervical cancer and HPV infection had higher levels of knowledge about HPV vaccination.^10^

In a cross-sectional study, in which Napolitoni et al. examined women’s knowledge and attitudes regarding HPV infection and vaccines, a positive significant relationship was found between women carrying and/or knowing about HPV risk factors and their levels of knowledge and attitudes in relation to HPV vaccines.^27^

## CONCLUSION

In our study, women’s knowledge and attitudes towards cervical cancer and HPV infection were found to be inadequate. This inadequacy had an effect on their levels of knowledge regarding HPV vaccines. The highest score on the scale of knowledge about HPV vaccines was 56, but the mean score of these women was 33.37 ± 5.05. Through this result, it was seen that women did not have enough information about HPV vaccines and that HPV vaccine-related education was needed. It was also found that women were not getting vaccinated because of their lack of knowledge about vaccine access, its effects and its cost. Considering the efforts made towards ensuring widespread use of cervical cancer screening programs, similar strategies and programs need to be developed for HPV vaccine programs, in order to provide greater immunity against HPV infection. Further qualitative and quantitative studies are needed in order to determine HPV vaccine awareness in Turkey.
